# Updates to Spectrum's case surveillance and vital registration tool for HIV estimates and projections

**DOI:** 10.1002/jia2.25777

**Published:** 2021-09-21

**Authors:** Severin G. Mahiane, Jeffrey W. Eaton, Robert Glaubius, Kelsey K. Case, Keith M. Sabin, Kimberly Marsh

**Affiliations:** ^1^ Center for Modeling and Analysis Avenir Health Glastonbury Connecticut USA; ^2^ MRC Centre for Global Infectious Disease Analysis, School of Public Health Imperial College London London UK; ^3^ Strategic Information Department UNAIDS Geneva Switzerland

**Keywords:** HIV case‐based surveillance, HIV incidence, HIV‐related mortality, key population, knowledge of status, statistical models

## Abstract

**Introduction:**

The Case Surveillance and Vital Registration (CSAVR) model within Spectrum estimates HIV incidence trends from surveillance data on numbers of new HIV diagnoses and HIV‐related deaths. This article describes developments of the CSAVR tool to more flexibly model diagnosis rates over time, estimate incidence patterns by sex and age group and by key population group.

**Methods:**

We modelled HIV diagnosis rate trends as a mixture of three factors, including temporal and opportunistic infection components. The tool was expanded to estimate incidence rate ratios by sex and age for countries with disaggregated reporting of new HIV diagnoses and AIDS deaths, and to account for information on key populations such as men who have sex with men (MSM), males who inject drugs (MWID), female sex workers (FSW) and females who inject drugs (FWID). We used a Bayesian framework to calibrate the tool in 71 high‐income or low‐HIV burden countries.

**Results:**

Across countries, an estimated median 89% (interquartile range [IQR]: 78%–96%) of HIV‐positive adults knew their status in 2019. Mean CD4 counts at diagnosis were stable over time, with a median of 456 cells/μl (IQR: 391–508) across countries in 2019. In European countries reporting new HIV diagnoses among key populations, median estimated proportions of males that are MSM and MWID was 1.3% (IQR: 0.9%–2.0%) and 0.56% (IQR: 0.51%–0.64%), respectively. The median estimated proportions of females that are FSW and FWID were 0.36% (IQR: 0.27%–0.45%) and 0.14 (IQR: 0.13%–0.15%), respectively. HIV incidence per 100 person‐years increased among MSM, with median estimates reaching 0.43 (IQR: 0.29–1.73) in 2019, but remained stable in MWID, FSW and FWID, at around 0.12 (IQR: 0.04–1.9), 0.09 (IQR: 0.06–0.69) and 0.13% (IQR: 0.08%–0.91%) in 2019, respectively. Knowledge of HIV status among HIV‐positive adults gradually increased since the early 1990s to exceed 75% in more than 75% of countries in 2019 among each key population.

**Conclusions:**

CSAVR offers an approach to using routine surveillance and vital registration data to estimate and project trends in both HIV incidence and knowledge of HIV status.

## 1 INTRODUCTION

The Joint United Nations Programme on HIV/AIDS (UNAIDS)‐supported Spectrum software package is used by most countries in the world to generate annual estimates of HIV burden and monitor the HIV epidemic [1, 2, 3]. The specific modelling approaches utilized depend on the type of surveillance data available and the characteristics of the HIV epidemic. In most high‐ and middle‐income countries with relatively low‐level HIV epidemics, the most consistently available data are reported numbers of new HIV diagnoses (recorded through case surveillance) and HIV‐related deaths (recorded through vital registration). The Case Surveillance and Vital Registration (CSAVR) tool, part of the Spectrum suite, uses these data to estimate trends in new HIV infections and HIV diagnosis rates consistent with these data, accounting for HIV disease progression and impacts of antiretroviral therapy (ART) on survival [1, 4]. The model outputs estimate the number of new HIV infections, the number of people living with HIV (PLHIV), and the proportion of PLHIV who know their HIV status.

Previously described CSAVR methods for relating new diagnoses to incidence had some limitations [1]. First, diagnosis rates depended only on HIV‐related mortality rates and were assumed to increase over time. Second, estimates of HIV status awareness were not stratified by sex, CD4 count, or age. These strata are important for assessing the impact of interventions to monitor the HIV epidemic. The tool estimates incidence in the age group 15–49, which Spectrum then disaggregates using incidence rate ratios by sex and age estimated from published studies [5]. However, these incidence patterns may not generalize to some countries.

To more flexibly model diagnosis rates among PLHIV, we developed CSAVR to adopt a similar structure for modelling HIV diagnosis as the Shiny90 model, developed by Maheu‐Giroux et al. [6] for countries in sub‐Saharan Africa, to reconstruct rates of HIV testing and diagnosis over time to estimate the proportion of PLHIV who are aware of their HIV status [7]. Additionally, we expanded CSAVR to estimate the pattern of new infections by sex and age using disaggregated reporting of new HIV diagnoses and AIDS deaths. Finally, we proposed a model for estimating new infections among four key populations—men who have sex with men (MSM), males who inject drugs (MWID), female sex workers (FSW) and females who inject drugs (FWID) — using case surveillance data on probable transmission mode.

## 2 METHODS

CSAVR is a multistate model representing the adult population aged 15 years and older, stratified sex and single year of age. Modelled processes are HIV infection, HIV diagnosis, HIV disease progression through infection stages, AIDS death, ART initiation and effects of ART on survival. Assumptions about disease progression, AIDS mortality, ART initiation and ART impact are taken from Spectrum. The model estimates rates of HIV incidence and HIV diagnosis, from which other model outputs are simulated.

### 2.1 Data

Countries using CSAVR rely upon data from national HIV case surveillance and vital registration. Both HIV‐related deaths and new HIV diagnoses are stratified by sex and five‐year age groups. In the applications, we used new HIV diagnoses as reported by countries to UNAIDS and HIV‐related deaths adjusted for incompleteness and misclassification from the Global Burden of Disease (GBD) study or the World Health Organization [1].

### 2.2 HIV incidence

Similar to previous versions [1], CSAVR offers four different options for modelling the HIV incidence rate over time in the 15–49 age group: (a) a double‐logistic function, (b) a single‐logistic function, (c) a segmented polynomial or spline and (d) *r*‐logistic transmission model; details on the parameterization are in the Appendix S1. Prespecified incidence rate ratios distribute the age 15–49 incidence to incidence rates by sex and 5‐year age groups, similar to the approach used by Spectrum [8].

### 2.3 New HIV diagnoses

**Figure 1:**
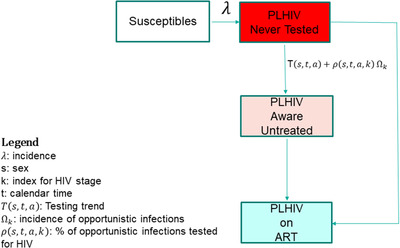
Intercompartmental flow describing diagnosis uptake. All parameters related to HIV diagnosis and incidence are estimated by the model. Other rates, such as antiretroviral therapy (ART) recruitment (adjusted for ART discontinuation), demographic parameters governing entry in the model at 15 years of age and both natural and HIV‐related mortality are informed by Spectrum.

We adapted the diagnosis model from Shiny90 to simulate diagnoses in HIV‐positive adults [6]. The 15–49 HIV‐positive population is stratified into three compartments: PLHIV who are unaware of their HIV status, status‐aware but not on ART, or status‐aware and on ART (Figure 1). Individuals enter the population at age 15 and are assumed to have never been tested for HIV (unless living with HIV and on ART). The model structure is the same as the EPP‐ASM model [9], a simplified version of Spectrum's AIDS Impact Module, which calculates population‐level HIV indicators from input HIV incidence trends [2, 9].

#### 2.3.1 Model specification

The per capita rate at which HIV‐positive individuals are diagnosed with HIV varies by sex, calendar time, age and CD4+ category. In our formulation, the diagnosis trend is a mixture of three factors: a Gaussian initial pulse, a logistic time trend and an opportunistic infections trend. The magnitudes of the initial and long‐term trends are controlled by two parameters, while five parameters control the rate of decrease and/or increase of the trends, the sex‐specific and opportunistic infections effects. We specify independent prior distributions on each parameter as specified in the Appendix S1.

### 2.4 Adjusting incidence rate ratios by sex and age

For countries with data on HIV diagnoses and HIV‐related deaths reported by age and sex, time‐varying sex ratios and age patterns of incidence are estimated. The female‐to‐male incidence rate ratio changes over time following a spline curve. HIV incidence rate ratios by age are proportional to a logit normal probability distribution over age with time‐varying mean and variance. Details on the parameterization and sample shapes of the incidence ratios that can be generated by this parameterization are given in Appendix S1.

### 2.5 Key population stratification

#### 2.5.1 Key populations characteristics

We considered four key populations: MSM, MWID, FSW and FWID, and assumed the proportion of adults who are in each key population are fixed over time. We assumed weak prior distributions on each key population size, informed by published studies [10, 11, 12, 13, 14, 15]. These size parameters are fitted to country‐specific input proportions specified by Spectrum users, when available.

Key population age distributions are assumed to be the same as the general population of the same sex at the beginning of the epidemic, but may change over time due to entry into and exit from the key population, termed “turnover.” Turnover rates can be inputted annually and for each key population except MSM. Recruitment rates adjust dynamically so that the proportion of adults in each key population remains constant over time.

#### 2.5.2 Modelling HIV incidence and new HIV diagnoses among key populations

We assumed the ratio of the incidence in each key population to the incidence of people with the same sex follows a time‐varying sigmoid function. Similarly, for each key population we assumed the diagnosis rate unrelated to opportunistic infections is proportional to the corresponding diagnosis rate in the general population of the same sex. These are simplifying assumptions that help reduce the number of parameters to be estimated.

We fitted the model with key populations for countries that reported data on source of HIV exposure recorded in The European Surveillance System (TESSy), a case surveillance reporting data system [16, 17, 18]. Published estimates of proportions of key populations and HIV prevalence among them were used to inform the priors [10, 11, 12, 13].

### 2.6 Model calibration

Model parameters were estimated using a Bayesian framework. Prior distributions and likelihood functions are described in the Appendix S1. Parameters determining the mode of the posterior distribution were optimized using a modified Nelder–Mead algorithm and Broyden–Fletcher–Goldfarb–Shanno algorithm [19, 20]. For each country, one of the four options for HIV incidence models was selected using Akaike Information Criterion (AIC, the lower the better) [21, 22]. Following selection of the incidence model, the full joint posterior distribution was estimated using an adaptive Markov chain Monte Carlo algorithm [23] starting from the mode estimated at the previous step. Uncertainty intervals for quantities of interest were obtained by sampling 1000 sets of parameter values, with 10,000 burn‐in samples and discarding 14 in 15 samples. The posterior distributions of relevant outputs were summarized using their mode and 2.5th and 97.5th percentiles.

The tool was developed as part of Spectrum [2, 3, 5]. However, analyses presented here were performed using an R implementation; all functions are available for download from the GitHub repository (https://github.com/guy2015/CSAVR).

#### 2.6.1 Model outputs

CSAVR generates outputs about the number of new HIV diagnoses by age, sex and CD4 categories. In addition, it estimates the proportion of PLHIV who know their status by age, sex and CD4 category. When key populations are added to the model, the results are further stratified by population group.

#### 2.6.2 Countries application

CSAVR was used to estimate and project HIV indicators in 71 countries from six UNAIDS regions (Table 1). The only inclusion criterion for countries was the availability of case surveillance data in their Spectrum/UNAIDS files. Countries in the regions of Asia and the Pacific (AP), eastern Europe and central Asia (EECA) and western and central Europe and North America (WCENA) more often have data for longer periods and disaggregated by sex. Only one country in the Caribbean region (CAR) had AIDS deaths disaggregated by sex. Fewer than 20% of countries in the Middle East and North Africa (MENA) and Latin America (LA) had reported new diagnoses by sex in any year since the epidemic began.

**Table 1:** List of countries included in the analysis

Asia and Pacific (AP)	Caribbean (CAR)	Eastern Europe and central Asia (EECA)	Latin America (LA)	Middle East and North Africa (MENA)	Western and central Europe and North America (WCENA)
Australia	Bahamas	Armenia^a^	Argentina	Algeria	Austria^a^
Japan	Barbados	Belarus^a^	Brazil	Egypt	Belgium^a^
New Zealand	Cuba	Georgia^a^	Chile	Jordan	Bulgaria^a^
Republic of Korea	Trinidad and Tobago	Kazakhstan	Colombia	Kuwait	Croatia^a^
Singapore		Kyrgyzstan^a^	Costa Rica	Lebanon	Cyprus^a^
		Republic of Moldova	El Salvador	Oman	Czech Republic^a^
		North Macedonia	Mexico	Qatar	Denmark^a^
		Russian Federation	Panama	Saudi Arabia	Estonia^a^
		Uzbekistan^a^	Uruguay	United Arab Emirates	Finland^a^
			Venezuela		France^a^
					Germany^a^
					Greece^a^
					Hungary^a^
					Iceland^a^
					Ireland^a^
					Israel
					Italy^a^
					Latvia^a^
					Lithuania^a^
					Luxembourg^a^
					Malta^a^
					Netherlands^a^
					Norway^a^
					Poland^a^
					Portugal^a^
					Romania^a^
					Serbia^a^
					Slovakia^a^
					Slovenia^a^
					Spain^a^
					Sweden^a^
					Switzerland^a^
					Turkey^a^
					United Kingdom^a^
					United States of America

^a^ Countries reporting new HIV diagnoses to The European Surveillance System (TESSy) and included in the analyses using key population data.

#### 2.6.3 Model validation

We validated the new version of CSAVR by performing out‐of‐sample predictions of both the yearly number of new diagnoses and AIDS deaths. The last 3 years of data were excluded from the estimation process and used to estimate the coverage probabilities. The continuous ranked probability score [24] and coverage probability were used to evaluate model performance.

## 3 RESULTS

**Figure 2:**
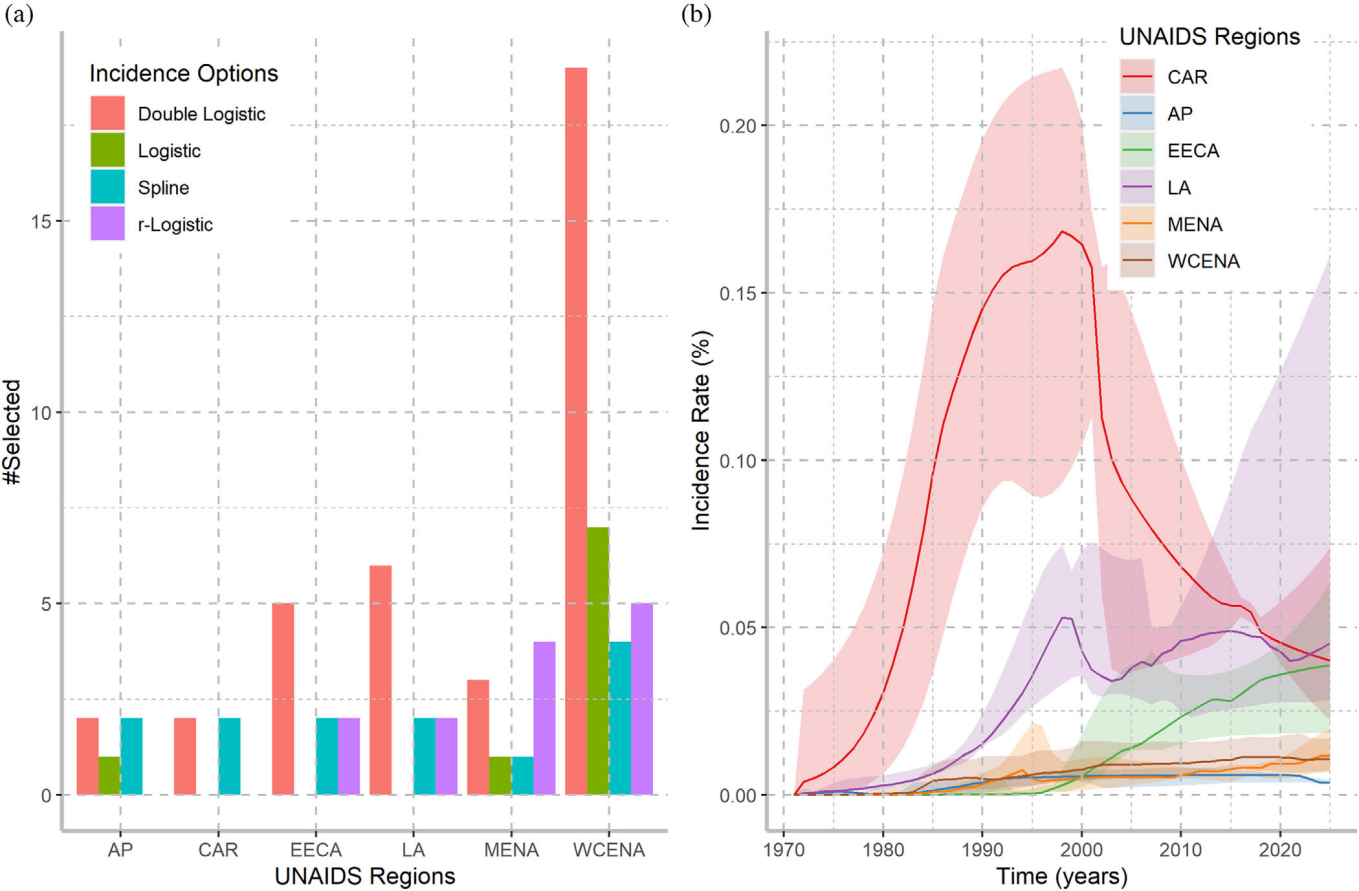
Selected incidence models and incidence rates among adults 15–49 years. **(a)** Number of countries using each incidence model. The *x*‐axis indicates the regions and the *y*‐axis indicates the number of times each incidence model was selected by region. **(b)** Median incidence rate (solid lines) and interquartile ranges (shaded regions) by UNAIDS region. The *x*‐axis indicates the calendar year and the *y*‐axis indicates incidence rate in person‐years expressed as percentage. AP, Asian Pacific; CAR, Caribbean; EECA, Eastern Europe and central Asia; LA, Latin America; MENA, Middle East and North Africa; WCENA, Western and central Europe and North America.

We fit the four incidence models (double logistic, single logistic, spline and *r*‐logistic) for each country. The best individual incidence model was selected using the AIC [21, 22]. Figure 2a,b displays the preferred incidence model by UNAIDS regions and the trends of their incidence rates. The double‐logistic model was the preferred model in EECA, LA and WCENA regions; the *r*‐logistic model was preferred in MENA. Overall, the AIC suggested that the double‐logistic model was the preferred model in most countries (51%), followed by the spline and *r*‐logistic models (18%). The median incidence was relatively stable from 2000 to 2019 in AP, WCENA and MENA. Incidence has slowly increased in LA and EECA. In the CAR, incidence peaked in the early 1990s and is currently decreasing. Overall, the median percentage of HIV‐positive people who knew their status was 89% (interquartile ranges [IQR]: 78%–96%) in 2019.

**Figure 3:**
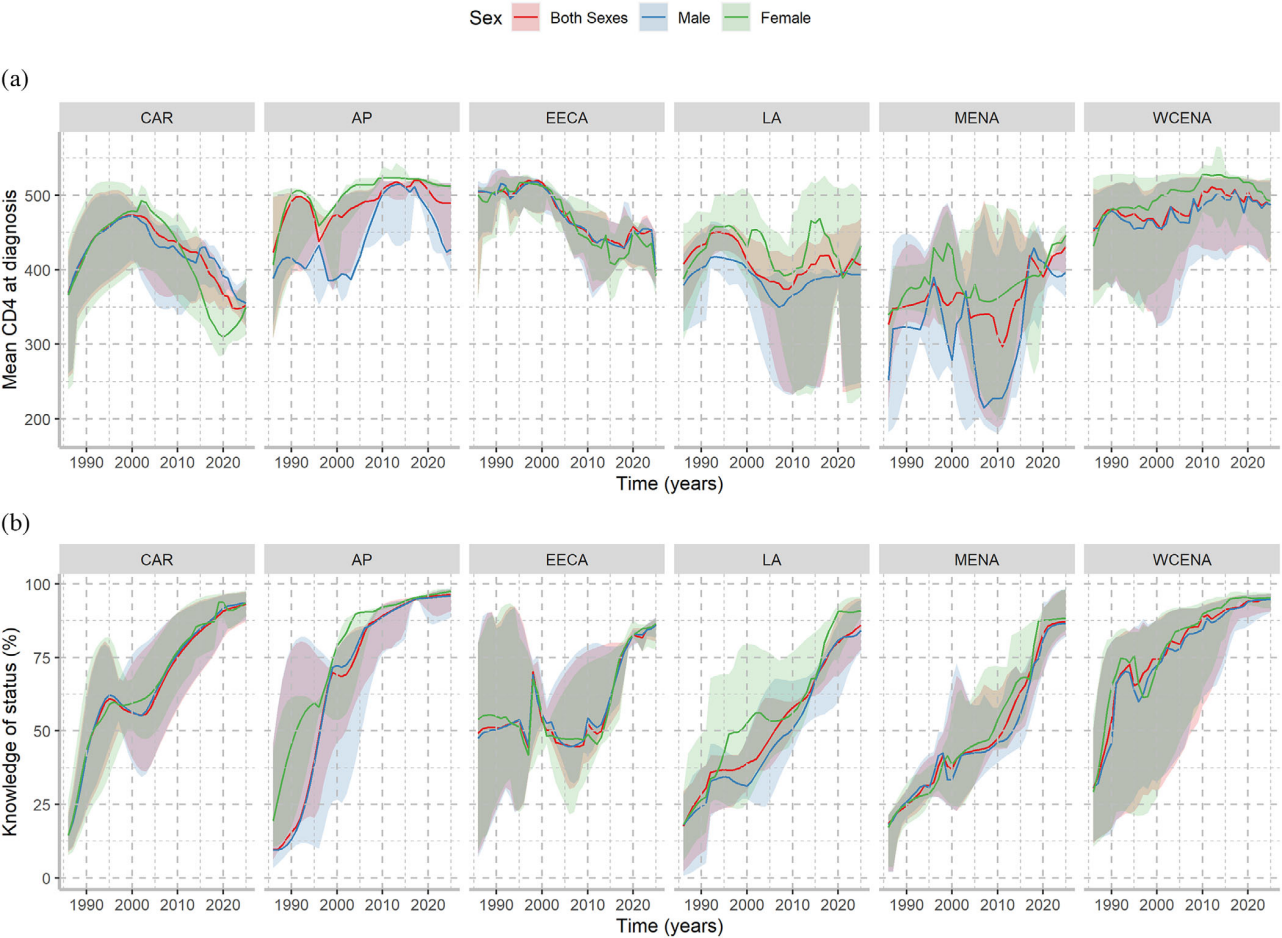
Mean CD4 at diagnosis and proportion of people living with human immunodeficiency virus (PLHIV) aware of their HIV status by sex and UNAIDS region. **(a)** Median and interquartile ranges (IQR) of mean CD4 at diagnosis. **(b)** Median and IQR of proportion of PLHIV aware of their HIV status. Each panel represents one of the UNAIDS region. The medians are represented with solid lines and IQR are represented by shaded regions. The *x*‐axes indicate the calendar year and the *y*‐axes indicate the mean CD4 at diagnosis (a) or knowledge of HIV status among HIV‐infected individuals (b). AP, Asian Pacific; CAR, Caribbean; EECA, Eastern Europe and central Asia; LA, Latin America; MENA, Middle East and North Africa; WCENA, Western and central Europe and North America.

Figure 3a displays the trends of the medians and IQR of mean CD4 counts at diagnosis across the regions. Medians ranged between 200 and 500 cells/μl in the mid‐1980s and stabilized above 450 cells/μl later. In 2019, across countries the median was 456 cells/μl (IQR: 391–508). CD4 at diagnosis was higher among women compared to men in MENA, LA and WCENA, while the value was similar for both sexes in EECA, CAR and AP. Figure 3b shows the trends of the estimated medians and IQR of the proportion of PLHIV who know their status. Awareness of status has increased among PLHIV in all the regions since the early 2000s, exceeding 75% in 2019 in more than 75% of countries in all regions. Knowledge of status was lowest for EECA, LA and MENA, where knowledge of status was greater among women living with HIV compared to men.

### 3.1 Out‐of‐sample predictions

According to the continuous ranked probability scores metric, the spline incidence model performed best in the out‐of‐sample prediction assessment in EECA and MENA, while the double‐logistic model was best in LA and WCENA (Appendix S1). The spline incidence model gave the best continuous ranked probability score in 35% of countries, followed by the double‐logistic (32%) and the *r*‐logistic (18%) incidence models.

The coverage probability of 95% posterior predictive intervals over the 3 years was very low, with the mean below 20% in AP, for all the incidence models. The single‐logistic model had the worst performance in CAR, EECA, MENA, LA and WCENA, with means of about 15%, 10%, 20%, 35% and 27%, respectively. The maximum mean coverage was about 55%, 50%, 40%, 50% and 30% in CAR, EECA, LA, MENA and WCENA, respectively, and was obtained by either the spline (CAR, LA, MENA) or the *r*‐logistic models. The coverages were higher when restricted to 1‐year prediction and calculated for the models selected by the AIC by approximately 55% (60% for AIDS deaths and 50% for new diagnoses).

### 3.2 Model with key populations

**Figure 4:**
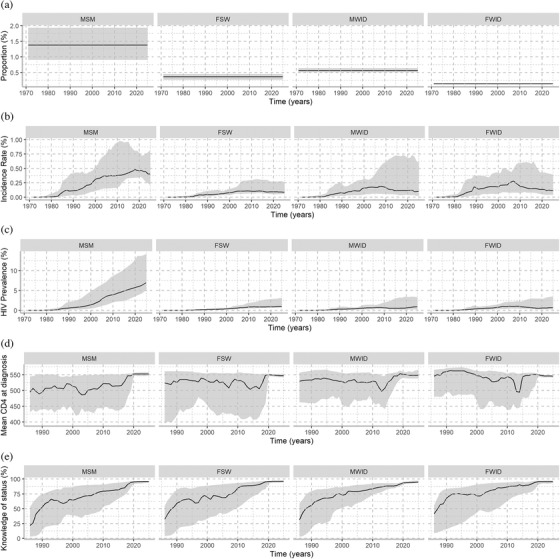
Estimated and projected trends of indicators in Europe. (a) proportions of key populations; (b) HIV incidence; (c) HIV prevalence; (d) mean CD4 at diagnosis among these populations; (e) knowledge of HIV status for those living with HIV. Each panel represents a key population group. FSW, female sex workers; FWID, females who inject drugs; MSM, men who have sex with men; MWID, males who inject drugs. The *x*‐axes indicate calendar year, and *y*‐axes indicate the indicator of interest. The solid lines represent the median across countries and the greyed areas indicate the interquartile ranges. Only countries reporting to The European Surveillance System (TESSy) were included in the analysis.

All four incidence functions were fit for each country and AIC was used to choose the best fit. Figure 4a, b, c, and d display the estimated and projected proportions of key populations, incidence, prevalence, mean CD4 at diagnosis and knowledge of HIV status among key populations living with HIV, respectively, in European countries included in the analysis. The median estimated proportions of males who were MSM and MWID were 1.3% (IQR: 0.9%–2.0%) and 0.56% (IQR: 0.51%–0.64%), respectively. The median estimated proportions of females who were FSW and FWID were 0.36% (IQR: 0.27%–0.45%) and 0.14 (IQR: 0.13%–0.15%), respectively. HIV incidence per 100 person‐years has slightly increased among MSM in recent years, with a median of 0.43 (IQR: 0.29–1.73) in 2019, but remained stable in MWID, FSW and FWID at around 0.12 (IQR: 0.04–1.9), 0.09 (IQR: 0.06–0.69) and 0.13 (IQR: 0.08–0.91) in 2019, respectively. The mean CD4 at diagnosis was slightly lower in FSW. Knowledge of status has gradually increased since the early 1990s to exceed 75% in more than 75% of countries, for each key population.

## 4 DISCUSSION

We described substantial development of Spectrum's CSAVR tool to model the HIV diagnosis rate more flexibly over time. The tool was first developed in the mid‐2010s to allow estimates and projection of the HIV epidemic in countries without sufficient HIV prevalence data from nationally representative surveys, but rather reliable records of case surveillance (i.e., new HIV diagnosis and HIV‐related deaths). The initial version made relatively strong assumptions regarding diagnosis trends and deaths rates and did not provide disaggregated estimates [1, 4].

The updated model allows estimating key indicators by age and sex. Furthermore, the model has also been expanded to fit data and provide estimates for key populations. However, high‐quality case surveillance data, consisting of relatively long and completed time series of surveillance data, are needed for the model to provide reliable estimates. Our analysis was restricted to countries that have reported these data to UNAIDS through previous applications of CSAVR. Therefore, our summary estimates can neither represent global nor regional estimates.

The elaboration of the new HIV diagnosis rate model was motivated by the Shiny90 model, used to estimate HIV testing rates in sub‐Saharan Africa. However, the implementation of the diagnosis rate in CSAVR differs from Shiny90 in many aspects. The Shiny90 diagnosis model accounts for testing in the HIV‐negative population and retesting, leveraging the national population‐based surveys and HIV testing data available. This component is not incorporated in CSAVR because the model was designed for use in countries where these data are not available. Furthermore, Shiny90 builds on prior information available in the literature to model diagnosis rate as a function of both age and sex. Many countries using CSAVR do not disaggregate new diagnoses by sex in their Spectrum files. In addition, Shiny90 assumes that diagnosis rates are negligible before 1995, whereas many countries that use CSAVR reported new HIV diagnoses in earlier years.

Countries that experienced substantial disruptions on their healthcare services due to COVID‐19 may find it difficult to fit their 2021 data with the current version of the tool. The two main components of our diagnosis trend capture diagnosis due to opportunistic infections and diagnosis unrelated to opportunistic infections. The functional form for diagnosis not due to opportunistic infections is a continuous function, thus it may not accommodate big jumps due to disruptions in HIV testing services, although changes in ART coverage will be accounted for. This could be resolved in future updates by allowing countries to specify reductions in services, which will translate in reductions of the diagnosis trends.

There are some key challenges faced when using the tool that still need to be addressed. The estimation process does not account for the quality and completeness of program data inputs or CD4 count measurement at diagnosis. This needs to be modelled, but was left for future work. The likelihood function only allows synthesis of information from case surveillance and vital registration. Improvements are needed to account for new diagnoses data from other sources, including among children, antenatal care, national HIV surveys and/or testing campaigns, when available. These adjustments to CSAVR are future priorities.

The estimated coverage probabilities over 3‐year projections were low, but increased when restricting the prediction to 1 year or when using the continuous ranked probability score for model selection. This indicates the weakness of CSAVR for medium‐ or long‐term predictions and indicates that there are likely unaccounted heterogeneities in these data.

We assumed that key population distributions and their characteristics such as turnover rates remained constant over time. This is a simplifying assumption, as those parameters might be time varying. The model can easily be adapted to account for this, though it would require further increasing the already large number of parameters, which may increase the time needed for model fitting. Furthermore, the key population‐extended model will only be relevant for countries with routine surveillance among key populations.

We made the simplifying assumption that the testing trends among key populations were proportional to the equivalent trend in the general population of the same sex. This is a limitation, as the true relationship is likely more complex. For example, the assumption is violated when targeted testing campaigns are implemented. However, the effect of this could be minimal if the epidemic is concentrated in one group, for example. More complex relations could express incidence trends among key populations as a function of incidence in the general populations. Also, the source of infection was self‐reported. This makes our estimates subject to bias, because some individuals may not disclose their behaviour or might have acquired HIV before or after they joined a key population group. Nevertheless, our size estimates are generally within the range of published estimates in Europe. For example, our overall median size of MSM is in the range 0.03%–5.6% found in Europe [14]; similarly, our median size estimate of MWID is between 0.49% and 2.0% estimated in western and eastern Europe, respectively [10], while our median size estimates of FWID is slightly lower than 0.2% and 0.64% estimated in western and eastern Europe, respectively [10]; and our median size estimate of FSW is between 0.4% and 0.6% reported in western and eastern Europe, respectively. However, discrepancies between our estimates and published values occur in some countries. In fact, our approach combines available information on size estimates, HIV prevalence and new HIV diagnoses in key populations. Divergence may occur if information in all those sources is not compatible or subject to potential biases.

Lastly, transgender populations were not modelled. The model was primarily developed to analyze data from TESSy database, which does not record data on transgender identity. Size estimates for FSW, MSM, people who inject drugs and transgender women are increasingly available, but quality varies widely [15]. Refinements of the approach will be needed to incorporate transgender individuals when more reliable estimates of the size and number of new diagnoses among this group become available.

Unlike the AIDS Epidemic Model [25], our model with key populations does not track the source of infections and is limited when it comes to evaluating HIV prevention interventions. Moreover, individuals are not tracked after they “leave” key population compartments (e.g., stop injecting drugs). This would require increasing the number of compartments of the model, which will make it more complex and increase simulation time. The main advantage of our approach is that model assumptions for the general population do not need to be changed when switching to the model without key populations.

Another area remaining for future work is to explore how countries might produce HIV incidence curves within smaller geographic areas using this tool. This would require countries to introduce changes in case notification forms to collect relevant information [1].

## 5 CONCLUSIONS

The CSAVR model has been improved and offers an approach to using routine surveillance and vital registration data to estimate and project trends in both HIV incidence and knowledge of HIV status, including in key populations.

## FUNDING

JWE was supported by UNAIDS, the Bill and Melinda Gates Foundation (OPP1190661), National Institute of Allergy and Infectious Disease of the National Institutes of Health under award number R01AI136664, and the MRC Centre for Global Infectious Disease Analysis (reference MR/R015600/1), jointly funded by the UK Medical Research Council (MRC) and the UK Foreign, Commonwealth & Development Office (FCDO), under the MRC/FCDO Concordat agreement and is also part of the EDCTP2 programme supported by the European Union.

## DISCLAIMER

The views and opinions of the authors expressed herein do not necessarily state or reflect those of ECDC. The accuracy of the authors’ statistical analysis and the findings they report are not the responsibility of ECDC. ECDC is not responsible for conclusions or opinions drawn from the data provided. ECDC is not responsible for the correctness of the data and for data management, data merging and data collation after provision of the data. ECDC shall not be held liable for improper or incorrect use of the data.

## COMPETING INTERESTS

The authors declare that they have no competing interests.

## AUTHORS' CONTRIBUTIONS

Conceived, designed and performed the experiments: SGM, RG, KM, JWE and KMS. Analyzed the data: SGM, KM, RG and JWE. Wrote the first draft of the article: SGM, RB, and KM. Contributed to the writing of the article: SGM, JWE, RG, KM and KKC. Agree with the article's result and conclusions: SGM, JWE, RG, KM and KKC.

## Supporting information

**Appendix S1**. Description of models’ parameters likelihood formulations and sample results.
