# Supplementary material for: Updates to Spectrum's case surveillance and vital registration tool for HIV estimates and projections
Source: J Int AIDS Soc. 2021 Sep 21;24(Suppl 5):e25777. doi: 10.1002/jia2.25777 (PMC8454676; doi:10.1002/jia2.25777)
Supplement: Supplementary file 1 — Appendix S1. Description of models’ parameters likelihood formulations and sample results. [file JIA2-24-e25777-s001.docx]

**Supporting Information S1 for “Updates to Spectrum’s Case Surveillance and Vital Registration tool for the UNAIDS 2021 estimates”**

Severin Guy Mahiane^1§^, Jeffrey W. Eaton^2^, Robert Glaubius^1^, Kelsey K. Case^2^, Keith Sabin^3^, Kimberly Marsh^3^

^1^ Center for Modeling and Analysis, Avenir Health, Glastonbury, Connecticut, USA

^2^ MRC Centre for Global Infectious Disease Analysis, School of Public Health, Imperial College London, London, UK

^3^Strategic Information Department, UNAIDS, Geneva, Switzerland

^§^Correspondence to Severin Guy Mahiane, Center for Modeling and Analysis, Avenir Health, 655 Winding Brook Dr Suite 4040, Glastonbury, CT 06033, USA.

E-mail addresses of authors:

SGM: [gmahiane@avenirhealth.org](mailto:gmahiane@avenirhealth.org)

JWE: [jeffrey.eaton@imperial.ac.uk](mailto:jeffrey.eaton@imperial.ac.uk)

RG: [rglaubius@avenirhealth.org](mailto:rglaubius@avenirhealth.org)

KKC: k.case@imperial.ac.uk

KS: [SabinK@unaids.org](mailto:SabinK@unaids.org)

KM: marshkima@gmail.com

#### 1-Incidence options

Incidence options available in CSAVR have been detailed in ^[^[^1^](#_ENREF_1)^,^ [^2^](#_ENREF_2)^]^. These parametric family considered are as follows.

**Double logistic**

The double logistic incidence model is defined by the function:

|  | $\lambda\left( t,\boldsymbol{\theta}^{\boldsymbol{1}} \right)=\left( \frac{\exp\left( \alpha\left( t-t_{0} \right) \right)}{1+\exp\left( \alpha\left( t-t_{0} \right) \right)} \right)^{\frac{1}{\kappa_{1}}}\left( 2a\left( \frac{\exp\left( -\beta\left( t-t_{0} \right) \right)}{1+\exp\left( -\beta\left( t-t_{0} \right) \right)} \right)^{\frac{1}{\kappa_{2}}}+b \right)$ | Eq. 1 |
| --- | --- | --- |

with $\boldsymbol{\theta}^{\boldsymbol{1}}\boldsymbol{=}\left( \alpha,\beta,a,b,t_{0},\kappa_{1},\kappa_{2} \right)$ and $\alpha,\beta,a,b,t_{0},\kappa_{1},\kappa_{2}>0$.

|  | $\log\left( \alpha\right.)\sim N\left( -0.69,2 \right), \log(\beta)\sim N\left( -0.69,2 \right), t_{0}\sim logitN\left( -0.96,0.78,0.5,60 \right),$ | Eq. 2 |
| --- | --- | --- |
|  | $\log(-\log(a))\sim N\left( 2.44,2 \right), \log(-\log(b))\sim N\left( 1.95,2 \right), \log\left( \kappa_{1} \right),\log\left( \kappa_{2} \right) \sim N\left( 0,1 \right).$ |  |

This family of incidence function is a generalization of the family used in ^[^[^1^](#_ENREF_1)^,^ [^2^](#_ENREF_2)^]^. In fact, they coincide for$\kappa_{1}=\kappa_{2}=1$. We fit this model with the following prior distribution on its parameters:

**Single logistic**

For this option, incidence is defined by the function

|  | $\lambda\left( t,\boldsymbol{\theta}^{\boldsymbol{1}} \right)=\frac{\exp\left( -c+\alpha\left( t-\tilde{t}_{0} \right) \right)}{1+\exp\left( -c+\alpha\left( t-\tilde{t}_{0} \right) \right)}$ | Eq. 3 |
| --- | --- | --- |

where $\tilde{t}_{0}=1970,$ $\boldsymbol{\theta}^{\boldsymbol{1}}\boldsymbol{=}\left( c,\alpha\right)$ and $\alpha,c>0$.

We fitted this model with the following prior distributions on its parameters:

|  | $\log(c)\sim N\left( 3,1 \right), \alpha\sim N\left( -5,5 \right).$ | Eq. 4 |
| --- | --- | --- |

**Second order segmented polynomial (splines)**

Second order segmented polynomial functions were included as a more flexible alternative to the single and double logistic functions ^[^[^1^](#_ENREF_1)^]^. The number of knots was set to four and their positions are estimated. Because the spline is not naturally constrained to be positive, it was transformed as follows:

|  | $\lambda\left( t,\boldsymbol{\theta}^{\boldsymbol{1}} \right)=\lambda_{max}\frac{i^{2}(t)}{1+i^{2}(t)}$ | Eq. 5 |
| --- | --- | --- |

where $\lambda_{max}$ is the largest possible value allowed for the incidence rate,

$i\left( t \right)=a_{k-1}+b_{k-1}\left( t-t_{k-1} \right)+c_{k-1}{(t-t_{k-1})}^{2}$, for $t$ in$\left( t_{k-1},t_{k} \right)$, $t_{0}=1970$,

and $a_{k},b_{k}, k=0..4$ and $t_{k}, k=1..4$ are parameters to be estimated; i.e. $\boldsymbol{\theta}^{\boldsymbol{1}}\boldsymbol{=}\left( a_{0},b_{0},c_{0},\left( c_{k},\zeta_{k} \right)_{k=1..4} \right)$. The model is fitted with the following prior distribution on its parameters:

$a_{0}\sim N\left( -30,2 \right), b_{0}\sim N\left( -10,2 \right),$ $c_{k}\sim\left( -1 \right)^{k+1}N\left( 0.005,1 \right), for all k$and

|  | $\zeta_{k}\left( \frac{t_{k}-t_{k-1}}{t_{max}-t_{0}} \right)\sim N\left( \frac{0,1}{4} \right), \mathrm{for} k=1..4$ | Eq. 6 |
| --- | --- | --- |

where $t_{max}$ is the final year of the projection and $\zeta=(\zeta_{1},\zeta_{2},\zeta_{2},\zeta_{3})$ is the inverse of the transformation $\left( x_{1},x_{2},x_{3},x_{4} \right)\to\left( \frac{e^{x_{1}}}{1+\sum_{k=1}^{4} e^{x_{k}}},\frac{e^{x_{2}}}{1+\sum_{k=1}^{4} e^{x_{k}}},\frac{e^{x_{3}}}{1+\sum_{k=1}^{4} e^{x_{k}}},\frac{e^{x_{4}}}{1+\sum_{k=1}^{4} e^{x_{k}}} \right)$.

**r-logistic transmission model**

In this option, incidence rate is modelled through the transmission rate ^[^[^1^](#_ENREF_1)^,^ [^3^](#_ENREF_3)^]^. In fact, the incidence function is given by:

|  | $\lambda\left( t,\boldsymbol{\theta}^{\boldsymbol{1}} \right)=r\left( t \right)p\left( t \right)\left( 1-0.7\kappa\left( t \right) \right)$ | Eq. 7 |
| --- | --- | --- |

where $p\left( t \right)$ is the prevalence at time $t$, $\kappa$ is the ART coverage, and 0.7 is the average reduction in transmission per additional person on ART. We use a logistic function to model the logarithm of$r(t)$, termed *r-logistic* with four parameters:

|  | $\log\left( r\left( t \right) \right)=r_{0}-\left( r_{\infty}-r_{0} \right)\frac{1}{1+\exp\left( -\alpha\left( t-t_{mid} \right) \right)}$ | Eq. 8 |
| --- | --- | --- |

where $exp(r_{0})$ is the initial exponential growth rate of the epidemic, $exp(r_{\infty})$ is the equilibrium value for $r\left( t \right)$, $\alpha$ is the rate of change of $r\left( t \right)$ in the log-scale and $t_{mid}$ is the inflection point. For this model, we specify a fifth parameter, ι, as the incidence rate at time${t=t}_{0}$, providing the initial pulse of infections. This model is fitted with the following prior distributions on its parameters:

$$r_{0}\sim N\left( \log\left( 0.5 \right),0.5 \right), r_{\infty}\sim N\left( \log\left( 0.09 \right),0.3 \right), log(\alpha)\sim N\left( \log\left( 0.2 \right),0.5 \right),$$

|  | $t_{mid}\sim N\left( 1993, 5 \right), \iota\sim N\left( -13, 5 \right).$ | Eq. 9 |
| --- | --- | --- |

#### 2 Diagnosis parameters

The per capita rate $\tau(s,t,a,k)$ at which individuals are diagnosed for HIV varies by sex (s), calendar time (t), age (a) and CD4+ cell count k. More specifically, it takes the following form:

|  | $\begin{matrix} \tau\left( s,t,a,k \right)= & \left( \nu_{1}e^{-\left( \frac{t-t_{0}}{\sigma} \right)^{2}}+\nu_{2}\frac{e^{\alpha\left( t-t_{M} \right)}}{1+e^{\alpha\left( t-t_{M} \right)}} \right)r_{s}+\delta\rho\left( s,t,a,k \right)\Omega_{k} \\ & \end{matrix}$ | Eq. 10 |
| --- | --- | --- |

where $\nu_{1}\in\left[ 0,3 \right], \nu_{2}\in[0,10]$; $\sigma\in[0,30]$, $\alpha\in[0,1]$, and $t_{M}\in[t_{0},t_{max}+10]$, $t_{0}$is the year of first diagnosis and $t_{max}$is the last year of the projection, $\Omega_{k}$is the incidence of opportunistic infections, $\rho\left( s,t,a,k \right)$is the proportion of opportunistic infections, $\delta\in[0,1]$, $r\in[0,30]$ and $r_{s}=1$ for males, and $r_{s}=r$ for females; and each component of the parameter $\boldsymbol{\theta}^{\boldsymbol{2}}=\left( \nu_{1},\nu_{2},\sigma,\alpha, \delta,r,t_{M} \right)$ is estimated with independent prior following logit-normal distributions. More precisely, the model is fitted with the following prior distributions on its parameters:

|  | $\nu_{1}\sim\mathrm{Lo}\mathrm{git}N\left( 1,1.5,0,3 \right),$ $\nu_{2}\sim\mathrm{Lo}\mathrm{git}N\left( -4.3,2,0,7 \right),$ $\sigma\sim\mathrm{Lo}\mathrm{git}N\left( 1,1.5,0,45 \right)$ |  |
| --- | --- | --- |
|  | $\alpha\sim\mathrm{Lo}\mathrm{git}N\left( -0.96,0.78,0,1 \right),$ $\delta\sim\mathrm{Lo}\mathrm{git}N\left( 1,1.5,0,1 \right),$ $log(r)\sim N\left( 0,1 \right)$ |  |
|  | $t_{M}\sim\mathrm{Lo}\mathrm{git}N\left( -0.96,0.78,t_{0},t_{max}+10 \right)$ | Eq. 11 |

#### 3 Likelihood Function

The model estimates the following parameters$\boldsymbol{\theta}=\left( \boldsymbol{\theta}^{\boldsymbol{1}}\boldsymbol{,}\boldsymbol{\theta}^{\boldsymbol{2}} \right)$, where $\boldsymbol{\theta}^{\boldsymbol{1}}$is the component related to the selected functional form for incidence (i.e., the parameters determining the shape of the single or double logistic, segmented polynomials, or r-logistic models) and $\boldsymbol{\theta}^{\boldsymbol{2}}$ is the component related to the diagnosis rate.

The data consist of the number of new HIV diagnoses reported in a calendar year and the number of AIDS deaths recorded in a calendar year among adults (age 15+ years). Where available, the number of diagnoses and AIDS deaths are stratified by sex or by sex and age groups 15-to-19, 20-to-24, 25-to-29, 30-to-34, 35-to-39, 40-to-44, 45-to-49, and 50+.

In mathematical notation, let the data consist of numbers of new diagnoses ($n_{i}\left( t_{ij} \right), j=1\ldots j_{i}$), AIDS deaths ($n_{d}\left( t_{dj} \right), j=1\ldots j_{d})$, where for any$j$, $n_{i}\left( t_{ij} \right)$ and $n_{d}\left( t_{dj} \right)$ are vectors such that:

|  | | $n_{i}\left( t_{ij} \right)=\left( n_{i,0}\left( t_{ij} \right),n_{i,1}\left( t_{ij} \right),n_{i,2}\left( t_{ij} \right),\left( n_{i,1}^{a}\left( t_{ij} \right) \right)_{a=1\ldots8},\left( n_{i,2}^{a}\left( t_{ij} \right) \right)_{a=1\ldots8} \right)$ | Eq. 12 | |
| --- | --- | --- | --- | --- |
|  | $n_{d}\left( t_{dj} \right)=\left( n_{d,0}\left( t_{dj} \right),n_{d,1}\left( t_{dj} \right),n_{d,2}\left( t_{dj} \right),\left( n_{d,1}^{a}\left( t_{dj} \right) \right)_{a=1\ldots8},\left( n_{d,2}^{a}\left( t_{dj} \right) \right)_{a=1\ldots8} \right)$ | | | Eq. 13 |

For each vector, the first component is the total number of new diagnoses or AIDS deaths, respectively, the second and third components are the number stratified by sex (male/female), and the four the and fifth components represent the numbers for each age group. Depending on the granularity of available data in a given year, the entries should follow conventions: 1) when either the second or third component is missing the remaining components are also missing 2) when the second and third components are not missing and the first component is the sum of the first and second 3) components of the fourth and fifth vectors are either all missing or non-missing and, 4) when they are not missing their sums equal the second and third component, respectively.

We assume that our data follow Gamma distributions. Maximizing the likelihood is equivalent to minimizing:

$$nllik\left( \boldsymbol{\theta} \right)=\sum_{u=i,d} \sum_{j=1}^{j_{u}} \left( \frac{n_{u,0}\left( t_{uj} \right)}{\varphi_{u}\left( \boldsymbol{\theta} \right)}-\left( \xi_{u}\left( t_{uj};\boldsymbol{\theta} \right)-1 \right)\log\left( n_{u,0}\left( t_{uj} \right) \right)+\xi_{u}\left( t_{uj}\boldsymbol{;\theta} \right)\log\left( \varphi_{u}\left( \boldsymbol{\theta} \right) \right)-log\Gamma\left( \xi_{u}\left( t_{uj};\boldsymbol{\theta} \right) \right)-\sum_{s=1,2} \left( \frac{n_{u,s}\left( t_{uj} \right)}{n_{u,0}\left( t_{uj} \right)}\log\left( \frac{\hat{n}_{u,s}\left( t_{uj};\boldsymbol{\theta} \right)}{\hat{n}_{u,0}\left( t_{uj};\boldsymbol{\theta} \right)} \right) \right)+\sum_{a=1\ldots7} \frac{n_{us}^{a}\left( t_{uj} \right)}{n_{u,s}\left( t_{uj} \right)}\log\left( \frac{\hat{n}_{us}^{a}\left( t_{uj};\boldsymbol{\theta} \right)}{\hat{n}_{u,s}\left( t_{uj};\boldsymbol{\theta} \right)} \right) \right)$$

Eq. 14

where ($\hat{n}_{i}\left( t_{ij};\boldsymbol{\theta} \right), j=1\ldots j_{i}$), and ($\hat{n}_{d}\left( t_{dj};\boldsymbol{\theta} \right), j=1\ldots j_{d})$ are respectively the number of new HIV diagnoses and AIDS deaths predicted by the model for the parameter value $\boldsymbol{\theta}$**,** and

$\varphi_{u}\left( \boldsymbol{\theta} \right)=1+\frac{\sum_{j=1}^{j_{u}} \left( \hat{n}_{u,0}\left( t_{uj}\boldsymbol{;\theta} \right)-n_{u,0}\left( t_{uj} \right) \right)^{2}}{\sum_{j=1}^{j_{u}} \hat{n}_{u,0}\left( t_{uj}\boldsymbol{;\theta} \right)}$ and $\xi_{u}\left( t_{uj},\boldsymbol{\theta} \right)=1+\frac{\hat{n}_{u,0}\left( t_{uj}\boldsymbol{;\theta} \right)}{\varphi_{u}\left( \boldsymbol{\theta} \right)}$ , for $u=i,d$ and $j=1\ldots j_{u}$

We adjusted the parameters by maximizing the posterior distribution, which is equivalent to minimizing

|  | $L\left( \boldsymbol{\theta} \right)=-P_{1}\left( \boldsymbol{\theta}^{\boldsymbol{1}} \right)-P_{2}\left( \boldsymbol{\theta}^{\boldsymbol{2}} \right)+nllik\left( \boldsymbol{\theta} \right)$ | Eq. 15 |
| --- | --- | --- |

where $P_{1}$ is the log prior distribution for the incidence model parameters $\boldsymbol{\theta}^{\boldsymbol{1}}$determined by

(Eq. 2), (Eq. 4), (Eq. 6) or (Eq. 9), and $P_{2}$ is the log prior distribution for the diagnosis model parameters determined by (Eq. 11).

We estimated the maximum mode a posteriori using a modified Nelder-Mead (NM) algorithm and Broyden-Fletcher-Goldfarb-Shanno (BFGS) algorithm ^[^[^4^](#_ENREF_4)^,^ [^5^](#_ENREF_5)^]^. We first called NM, to move away from the starting parameter, in case it is not in the basin of attraction of the optimum; then BFGS is called to complete the optimization process.

We used the adaptive MCMC to estimate the join posterior distribution ^[^[^6^](#_ENREF_6)^]^, and uncertainty intervals for quantities of interest were obtained by sampling 1000, with 10000 burn-in samples and throwing 14 in 15 samples.

Akaike Information Criterion (AIC) ^[^[^7^](#_ENREF_7)^,^ [^8^](#_ENREF_8)^]^ was used for model selection. For each candidate model, $AIC\left( \boldsymbol{\theta} \right)=2 L\left( \boldsymbol{\theta} \right)+2 p$ where $p$ is the dimension of the parameter, was evaluated at the parameter minimizing (Eq. 15). Then, the model with the smallest AIC was chosen, i.e., estimates obtained using this latter model was used for estimations and projections.

#### 4 Incidence Rate Ratios

A tool was built within CSAVR to estimate incidence rate ratios (IRRs) for countries with new HIV diagnoses and AIDS deaths reported by age when estimates are not always specifically tailored to some countries or outdated. The model assumes that the female-to-male ratio is given by:

$$ftoM\left( t \right)=\theta_{0}^{3}+\left( \theta_{1}^{3}-\theta_{0}^{3} \right)\frac{e^{sp\left( t;\boldsymbol{\theta}_{2}^{\mathbf{3}} \right)}}{1+e^{sp\left( t;\boldsymbol{\theta}_{2}^{\mathbf{3}} \right)}}$$

where $\theta_{0}^{3}$ and $\theta_{1}^{3}$ are non-negative real numbers, $sp$ is a second order segmented polynomial (see Section 1) starting at 0 in 1970, with 3 knots to be estimated, and parameterized by $\boldsymbol{\theta}_{2}^{\mathbf{3}}$. The function $sp$ can be described by equation (Eq. 6) in which $k=0..3$ and the first component is dropped or set to zero. The female-to-male ratio parameter is thus parameterized by $\boldsymbol{\theta}^{\boldsymbol{3}}\boldsymbol{=}\left( \theta_{0}^{3},\theta_{1}^{3},\boldsymbol{\theta}_{2}^{\mathbf{3}} \right)$. The prior information on $\boldsymbol{\theta}^{\boldsymbol{3}}$ was specified as follows. $\log\left( \theta_{0}^{3} \right),\log\left( \theta_{1}^{3} \right)\sim N\left( -1.61,1 \right)$, and the prior of can be obtained by Eq. 6 in which $a_{0}$ is dropped and $k=0..3$. The log of the density of the prior distribution on this parameter will be denoted $P_{3}$.

The model also assumes that the age ratios vary as a function of sex, $s$, and time, $t$, and are given by:

$AgeR\left( a,s,t \right)=\left\{ \begin{matrix} g\left( a,\mu\left( s,t \right),d\left( s,t \right),15,49 \right) & if 15\leq a<49 \\ g\left( 49,\mu\left( s,t \right),d\left( s,t \right),15,49 \right)\left( \frac{80-a}{31} \right)^{2} & if 49\leq a\leq80 \\ 0 & if a>80 \end{matrix} \right.$

where $g$ is proportional to the density of a logit normal distribution with parameters $\mu\left( s,t \right)$ and $d\left( s,t \right)$ in the interval (15,49) and such that $g\left( 27.5,\mu\left( s,t \right),d\left( s,t \right),15,49 \right)=1$ for any $\mu\left( s,t \right)$ and for any $d\left( s,t \right)$, and

$\mu\left( s,t \right)=1-4\frac{e^{\theta_{0,s}^{4}+\theta_{1,s}^{4}\left| t-\check{t}\left( \theta_{2,s}^{4} \right) \right|^{\phi_{s}}}}{1+e^{\theta_{0,s}^{4}+\theta_{1,s}^{4}\left| t-\check{t}\left( \theta_{2,s}^{4} \right) \right|^{\phi_{s}}}}$, $d\left( s,t \right)=0.1-1.9\frac{e^{\theta_{4,s}^{4}+\theta_{5,s}^{4}\left| t-\check{t}\left( \theta_{2,s}^{4} \right) \right|^{\phi_{s}}}}{1+e^{\theta_{4,s}^{4}+\theta_{5,s}^{4}\left| t-\check{t}\left( \theta_{2,s}^{4} \right) \right|^{\phi_{s}}}}$

with $\check{t}\left( x \right)=50\frac{e^{x}}{1+e^{x}}$ for all x and $\phi_{s}=1-\frac{e^{\theta_{3,s}^{4}+ \theta_{6,s}^{4}\left( t-\check{t}\left( \theta_{2,s}^{4} \right) \right)}}{1+e^{\theta_{3,s}^{4}+ \theta_{6,s}^{4}\left( t-\check{t}\left( \theta_{2,s}^{4} \right) \right)}}$.

We parameterized the incidence rate ratios by $\boldsymbol{\theta}^{\boldsymbol{4}}\boldsymbol{=}\left( \theta_{j,s}^{4} \right)_{\boldsymbol{j=0\ldots7, s=1,2}}$.

In the meantime, the prior on $\boldsymbol{\theta}^{\boldsymbol{4}}$ is such that

|  | $\theta_{0,s}^{4}\sim N\left( -1.61,1 \right)$, $\theta_{1,s}^{4}\sim N\left( -1.61,1 \right)$, $\theta_{2,s}^{4}\sim N\left( -0.41,1 \right)$, $\theta_{3,s}^{4}\sim N\left( 4.5E-7,1 \right)$, |  |
| --- | --- | --- |
|  | $\theta_{4,s}^{4}\sim N\left( 0.5,1 \right)$, $\theta_{5,s}^{4}\sim N\left( -7.8E-6,1 \right)$, $\theta_{6,s}^{4}\sim N\left( -3.5E-6,1 \right)$ | Eq. 16 |

The prior on $\boldsymbol{\theta}^{\boldsymbol{4}}$, $P_{4}$, is determined by Eq. 16.

When there is enough data, incidence rate ratios as well as the overall incidence in the age group 15-to-49 parameters presented in Section 1 can be fitted simultaneously. In that case, the full parameter is

$\boldsymbol{\theta}^{\boldsymbol{'}}\boldsymbol{=}\left( \boldsymbol{\theta,}\boldsymbol{\theta}^{\boldsymbol{3}}\boldsymbol{,}\boldsymbol{\theta}^{\boldsymbol{4}} \right)$ and the objective function given by Eq. 15 is replaced with

|  | $L\left( \boldsymbol{\theta}^{\boldsymbol{'}} \right)=-P_{1}\left( \boldsymbol{\theta}^{\boldsymbol{1}} \right)-P_{2}\left( \boldsymbol{\theta}^{\boldsymbol{2}} \right)-P_{3}\left( \boldsymbol{\theta}^{\boldsymbol{3}} \right)-P_{4}\left( \boldsymbol{\theta}^{\boldsymbol{4}} \right)+nllik\left( \boldsymbol{\theta} \right)$ | Eq. 17 |
| --- | --- | --- |

#### 5 Case of key populations

We consider four key populations: men who have sex with men (MSM), men who inject drugs (MWID), female sex workers (FSW), and females who inject drugs (FWID). Throughout this section, we denote $l=1,2$ an index for key population types; $l=1$for MSM and FSW and $l=2$ for people who inject drugs.

**Key population characteristics**

We assume that the proportions of key populations ($p_{l}^{M}$ and$p_{l}^{F}$, $l=1,2$ for males and females respectively) are fixed over time. The following prior distributions are assumed for each key population.

|  | $p_{1}^{M}\sim\mathrm{Logit}N\left( -3.7,1.42,0,0.5 \right), p_{2}^{M}\sim\mathrm{Logit}N\left( -3.7,1.7,0,0.15 \right),$ | Eq. 18 |
| --- | --- | --- |

|  | $p_{1}^{F}\sim\mathrm{Logit}N\left( -2.9,1.4,0,0.15 \right), p_{2}^{F}\sim\mathrm{Logit}N\left( -3.76,1.7,0,0.15 \right)$. | Eq. 19 |
| --- | --- | --- |

These parameters are fitted to the observed proportions which are specified by users.

The age distribution of KPs at the beginning of the epidemic is assumed to be identical to that of the general population of the same sex. However, turnover rates, which are inputs to the model, are applied every year for all ages, for all KPs but MSM. This may result in a change of age distribution of KPs over time. Recruitment rates are adapted to maintain the proportion of the population belonging to each KPs constant.

**Incidence**

Let $\lambda_{M}$be the incidence rate among all men and $\lambda_{F}$ be the incidence rate among all females. Thus, for MSM and MWID, the incidence rate as a function of time is obtained by:

$$\lambda_{Ml}\left( t \right)=\lambda_{M}\left( \psi_{1l}^{M}+\left( \psi_{0l}^{M}-\psi_{1l}^{M} \right)e^{-\tilde{\psi}_{l}^{M}\left( t-t_{l}^{M} \right)} \right)$$

and for FSW and FWID, we have:

$$\lambda_{Fl}\left( t \right)=\lambda_{F}\left( \psi_{1l}^{F}+\left( \psi_{0l}^{F}-\psi_{1l}^{F} \right)e^{-\tilde{\psi}_{l}^{F}\left( t-t_{l}^{F} \right)} \right)$$

where $\psi_{ml}^{s}\geq0,$for $m=0,1$ and $s=M,F$, $\tilde{\psi}_{l}^{M}=\frac{1}{1+0.5\left( \psi_{0l}^{M}+\psi_{1l}^{M} \right)}$ and $\tilde{\psi}_{l}^{F}=\frac{1}{1+0.5\left( \psi_{0l}^{F}+\psi_{1l}^{F} \right)}$ .

The incidence parameter is thus augmented by$\boldsymbol{\theta}^{\boldsymbol{0}}\boldsymbol{=}\left( \psi_{0l}^{M}, \psi_{1l}^{M},t_{l}^{M},\psi_{0l}^{F},\psi_{0l}^{F},t_{l}^{F} \right)_{l=1,2}$. This parameter is fitted with the following prior information.

|  | $\psi_{01}^{M},\psi_{02}^{M},\psi_{01}^{F},\psi_{02}^{F}\sim\mathrm{Logit}N(-0.96,1.6,0,100)$, |  |
| --- | --- | --- |
|  | $t_{1}^{M},t_{2}^{M},t_{1}^{F},t_{2}^{F}\sim\mathrm{Logit}N(-0.96,1.6,0,60)$ | Eq. 20 |

**New HIV diagnoses**

We assume that, for each key population, that rate is scaled by a factor ($r_{l}^{M}$ and$r_{l}^{F}$, $l=1,2$ for males and females respectively) fixed over time. More specifically if we denote the rate at which key populations are tested by$\tau_{l}$, then we have

|  | $\tau_{l}\left( s,t,a,k \right)=r_{l}^{s}\left( \tau\left( s,t,a,k \right)-\delta\rho\left( s,t,a,k \right)\Omega_{k} \right)+\delta\rho\left( s,t,a,k \right)\Omega_{k}$ | Eq. 21 |
| --- | --- | --- |

The parameters $\left( r_{l}^{s} \right)_{\begin{aligned} s=F,M \\ l=1,2 \end{aligned}}$ are fitted with the following prior distribution.

|  | $log\left( r_{1}^{M} \right),\log\left( r_{2}^{M} \right),\log\left( r_{1}^{F} \right),\log\left( r_{2}^{F} \right)\sim N(0,1)$ | Eq. 22 |
| --- | --- | --- |

**Likelihood**

We still assume that data consist of numbers of new diagnoses ($n_{i}\left( t_{ij} \right), j=1\ldots j_{i}$), AIDS deaths ($n_{d}\left( t_{dj} \right), j=1\ldots j_{d})$ are given by (Eq. 12) and (Eq. 13). We further assume that, for key populations, the number of new diagnoses, and AIDS deaths are given by ($\eta_{i,s,l}\left( t_{ij} \right), j=1\ldots j_{i,s,l}$) and ($\eta_{d,s,l}\left( t_{dj} \right), j=1\ldots j_{d,s,l})$, where$s=M,F$,$l=1,2$ and for any j, $\eta_{i,s,l}\left( t_{ij} \right)$ and $\eta_{d,s,l}\left( t_{dj} \right)$are vectors such that:

|  | $\eta_{i,s,l}\left( t_{ij} \right)=\left( \eta_{i,s,l}^{0},\left( \eta_{i,s,l}^{a}\left( t_{ij} \right) \right)_{a=1\ldots8} \right)$ | Eq. 23 |
| --- | --- | --- |

|  | $\eta_{d,s,l}\left( t_{ij} \right)=\left( \eta_{d,s,l}^{0},\left( \eta_{d,s,l}^{a}\left( t_{dj} \right) \right)_{a=1\ldots8} \right)$ | Eq. 24 |
| --- | --- | --- |

and for each of vector, 1) when either the first component is missing the second component is also missing 2) components of the second component (which is a vector) are either all missing or non-missing and, 3) when they are not missing their sums equal the first. In fact, the first component represents the total numbers (of new HIV diagnoses or AIDS deaths), the second represents the numbers for each age group, where the age groups are 15-to-19, 20-to-24, 25-to-29, 30-to-34, 35-to-39, 40-to-44, 45-to-49, and 50+.

When key populations are added to the model, the model parameter is $\tilde{\boldsymbol{\theta}}\boldsymbol{=}\left( \boldsymbol{\theta}^{\boldsymbol{0}}\boldsymbol{,}\boldsymbol{\theta}^{\boldsymbol{1}}\boldsymbol{,}\boldsymbol{\theta}^{\boldsymbol{2}}\boldsymbol{,}\boldsymbol{\theta}^{\boldsymbol{5}} \right)\boldsymbol{=}\left( \boldsymbol{\theta}^{\boldsymbol{0}}\boldsymbol{,\theta,}\boldsymbol{\theta}^{\boldsymbol{5}} \right)$**,** where $\boldsymbol{\theta}^{\boldsymbol{0}}\boldsymbol{,}\boldsymbol{\theta}^{\boldsymbol{1}}\boldsymbol{,}\boldsymbol{\theta}^{\boldsymbol{2}}$ and $\boldsymbol{\theta}$ were defined in previous sections and $\boldsymbol{\theta}^{\boldsymbol{5}}\boldsymbol{=}\left( p_{1}^{M},p_{2}^{M}, p_{1}^{F}, p_{2}^{F}, r_{1}^{M},r_{2}^{M}, r_{1}^{F}, r_{2}^{F} \right)$.

The contribution of key populations to the negative log-likelihood is given by:

|  | $kp_{\_}nllik\left( \tilde{\boldsymbol{\theta}} \right)=\sum_{s=F,M} \sum_{l=1}^{2} \left( {m_{ls}p}_{l}^{s}-1 \right)\log\left( \hat{p}_{l}^{s}\left( \tilde{\boldsymbol{\theta}} \right) \right)+\left( m_{ls}\left( {1-p}_{l}^{s} \right)-1 \right)\log\left( 1-\hat{p}_{l}^{s}\left( \tilde{\boldsymbol{\theta}} \right) \right)+\sum_{s=F,M} \sum_{l=1}^{2} \sum_{u=i,d} \sum_{j=1}^{j_{u}} \left( \frac{\eta_{u,s,l}^{0}\left( t_{uj} \right)}{\varphi_{u,s,l}\left( \tilde{\boldsymbol{\theta}} \right)}-\left( \xi_{u,s,l}\left( t_{uj};\tilde{\boldsymbol{\theta}} \right)-1 \right)\log\left( \eta_{u,s,l}^{0}\left( t_{uj} \right) \right)+\xi_{u,s,l}\left( t_{uj};\tilde{\boldsymbol{\theta}} \right)\log\left( \varphi_{u,s,l}\left( \tilde{\boldsymbol{\theta}} \right) \right)-log\Gamma\left( \xi_{u,s,l}\left( t_{uj};\tilde{\boldsymbol{\theta}} \right) \right)-\sum_{s=F,M} \sum_{l=1}^{2} \sum_{a=1\ldots7} \frac{\eta_{u,s,l}^{a}\left( t_{uj} \right)}{\eta_{u,s,l}^{0}\left( t_{uj} \right)}\log\left( \frac{\hat{\eta}_{u,s,l}^{a}\left( t_{uj};\tilde{\boldsymbol{\theta}} \right)}{\hat{\eta}_{u,s,l}^{0}\left( t_{uj};\tilde{\boldsymbol{\theta}} \right)} \right) \right)$ | |  |
| --- | --- | --- | --- |
|  |  | Eq. 25 | |

where for all $s$ and $l$, $m_{ls}$ is the sample size used to estimate the size of the key population of interest and $\varphi_{u,s,l}\left( \tilde{\boldsymbol{\theta}} \right)=1+\frac{\sum_{j=1}^{j_{u}} \left( \hat{\eta}_{u,s,l}^{0}\left( t_{uj};\tilde{\boldsymbol{\theta}} \right)-\eta_{u,s,l}^{0}\left( t_{uj} \right) \right)^{2}}{\sum_{j=1}^{j_{u}} \hat{\eta}_{u,s,l}^{0}\left( t_{uj};\tilde{\boldsymbol{\theta}} \right)}$ and $\xi_{u}\left( t_{uj}\boldsymbol{;}\tilde{\boldsymbol{\theta}} \right)=1+\frac{\hat{\eta}_{u,s,l}^{0}\left( t_{uj};\tilde{\boldsymbol{\theta}} \right)}{\varphi_{u,s,l}\left( \tilde{\boldsymbol{\theta}} \right)}$ , for $u=i,d$ and $j=1\ldots j_{u}$. When $m_{ls}$ is missing, we use 500.

The estimation process of $\tilde{\boldsymbol{\theta}}$ was similar to the one used for$\boldsymbol{\theta}$**.** We minimized

|  | $\tilde{L}\left( \tilde{\boldsymbol{\theta}} \right)=-P_{0}\left( \boldsymbol{\theta}^{\boldsymbol{0}} \right)-P_{5}\left( \boldsymbol{\theta}^{\boldsymbol{5}} \right)+L\left( \boldsymbol{\theta} \right)+kp_{\_}nllik\left( \tilde{\boldsymbol{\theta}} \right)$ | Eq. 26 |
| --- | --- | --- |

where $P_{0}$ is the log prior distribution for the incidence model parameters $\boldsymbol{\theta}^{\boldsymbol{0}}$determined by Eq. 20 and $P_{3}$ is the log prior distribution for the combination of proportions of key populations and diagnosis model parameters $\boldsymbol{\theta}^{\boldsymbol{5}}$ determined by Eq. 18, Eq. 19 and Eq. 22, and is given $L\left( \boldsymbol{\theta} \right)$ by Eq. 15.

When there are enough data, incidence rate ratios, the overall incidence in the age group 15-to-49 parameters presented in Section 1, size of key populations and incidence and prevalence among them can be fitted simultaneously. In that case, the full parameter is ${\tilde{\boldsymbol{\theta}}}^{\boldsymbol{'}}\boldsymbol{=}\left( \boldsymbol{\theta}^{\boldsymbol{0}}\boldsymbol{,}\boldsymbol{\theta}^{\boldsymbol{1}}\boldsymbol{,}\boldsymbol{\theta}^{\boldsymbol{2}}\boldsymbol{,}\boldsymbol{\theta}^{\boldsymbol{3}}\boldsymbol{,}\boldsymbol{\theta}^{\boldsymbol{4}}\boldsymbol{,}\boldsymbol{\theta}^{\boldsymbol{5}} \right)$ and the objective function given by Eq. 26 is replaced with

|  | $\tilde{L}\left( {\tilde{\boldsymbol{\theta}}}^{\boldsymbol{'}} \right)=-P_{0}\left( \boldsymbol{\theta}^{\boldsymbol{0}} \right)-P_{5}\left( \boldsymbol{\theta}^{\boldsymbol{5}} \right)+L\left( \boldsymbol{\theta}^{\boldsymbol{'}} \right)+kp_{\_}nllik\left( \tilde{\boldsymbol{\theta}} \right)$ | Eq. 27 |
| --- | --- | --- |

where $\boldsymbol{\theta}^{\boldsymbol{'}}$is defined in Section4 and $L\left( \boldsymbol{\theta}^{\boldsymbol{'}} \right)$ is given by Eq. 17Eq. 16.

#### 6 Examples

**Application 1: CSAVR classic in Chile**

We ﬁtted the four incidence models (double logistic, single logistic, segmented polynomial, and r-logistic) for Chile, using 2019 files. Incidence rate ratios were adjusted for by all the options. The r-logistic model had the lowest AIC and was therefore used for projection. Figure S1 shows the estimated trends of sex age incidence rate ratios. It suggests that incidence has historically been higher among men although the gap has been reduced in recent years. More specifically, incidence varied from about 0.2 in the mid-1990s to about 0.85 in 2019. Age rate ratios have not changed much among men, with incidence reaching its peak between the aged 20 and 30. In contrast, the distribution of new infections by age among women appear to have changed and has become similar to men since the mid-2000s. Figure S2 displays reported and estimated numbers of new HIV diagnoses, AIDS deaths, and proportion of people living with HIV knowing their status, and mean CD4 at diagnosis. The model seems to be adjusting to the data well and indicates that the knowledge of status has been decreasing since 2010 and was close to 90% among both men and women. CD4 at diagnosis was about 400 in 2019 and slightly higher among women.

**Remark:** The drop in new diagnoses suggested by the model after the latest data point is caused by the sudden drop in ART coverage in the projection file.

**
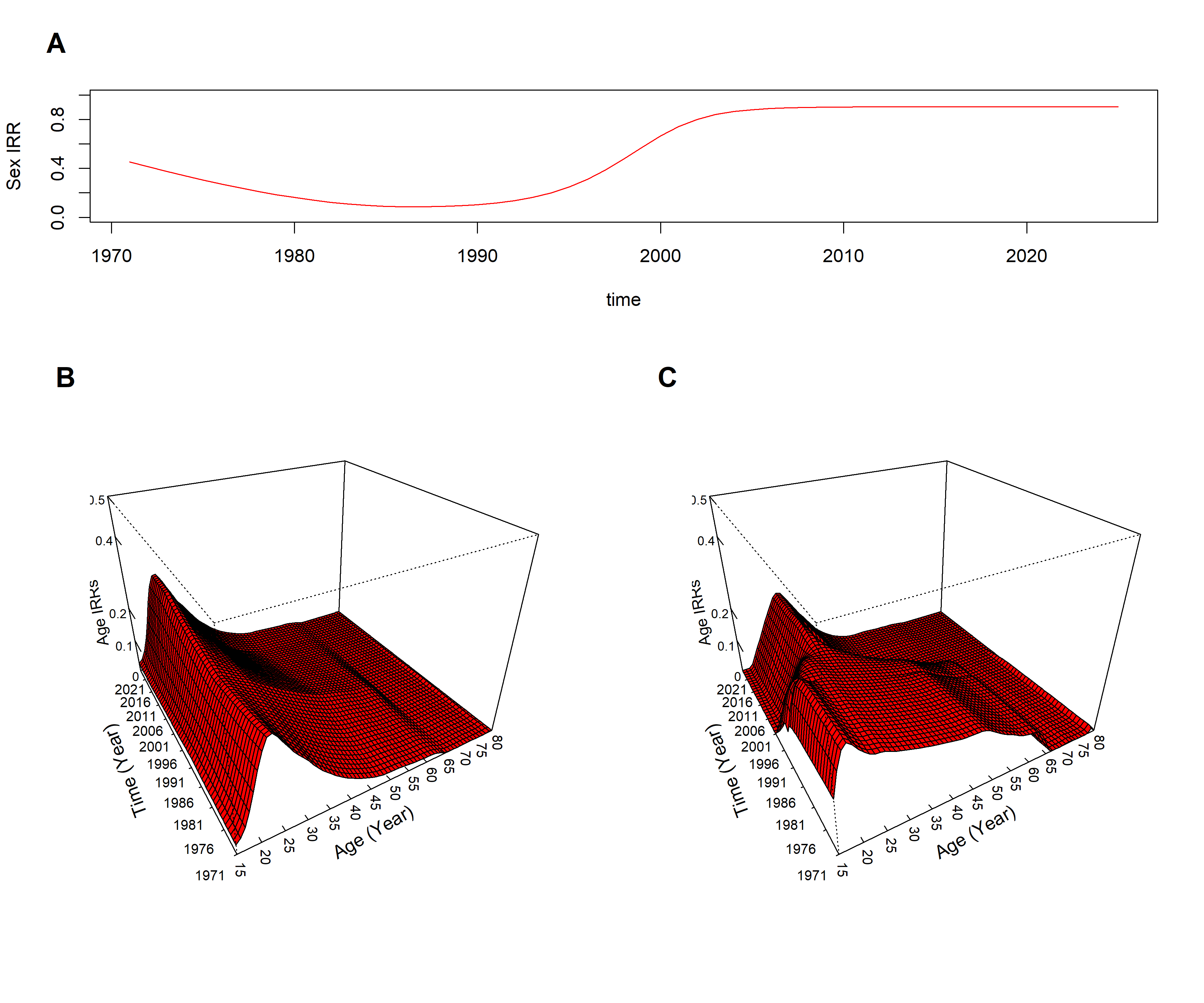
Figure S1:** Estimated trends of sex and age incidence rate ratios in Chile. A) Female to male incidence rate ratios surface for men, B) age incidence rate ratios surface for women.

**
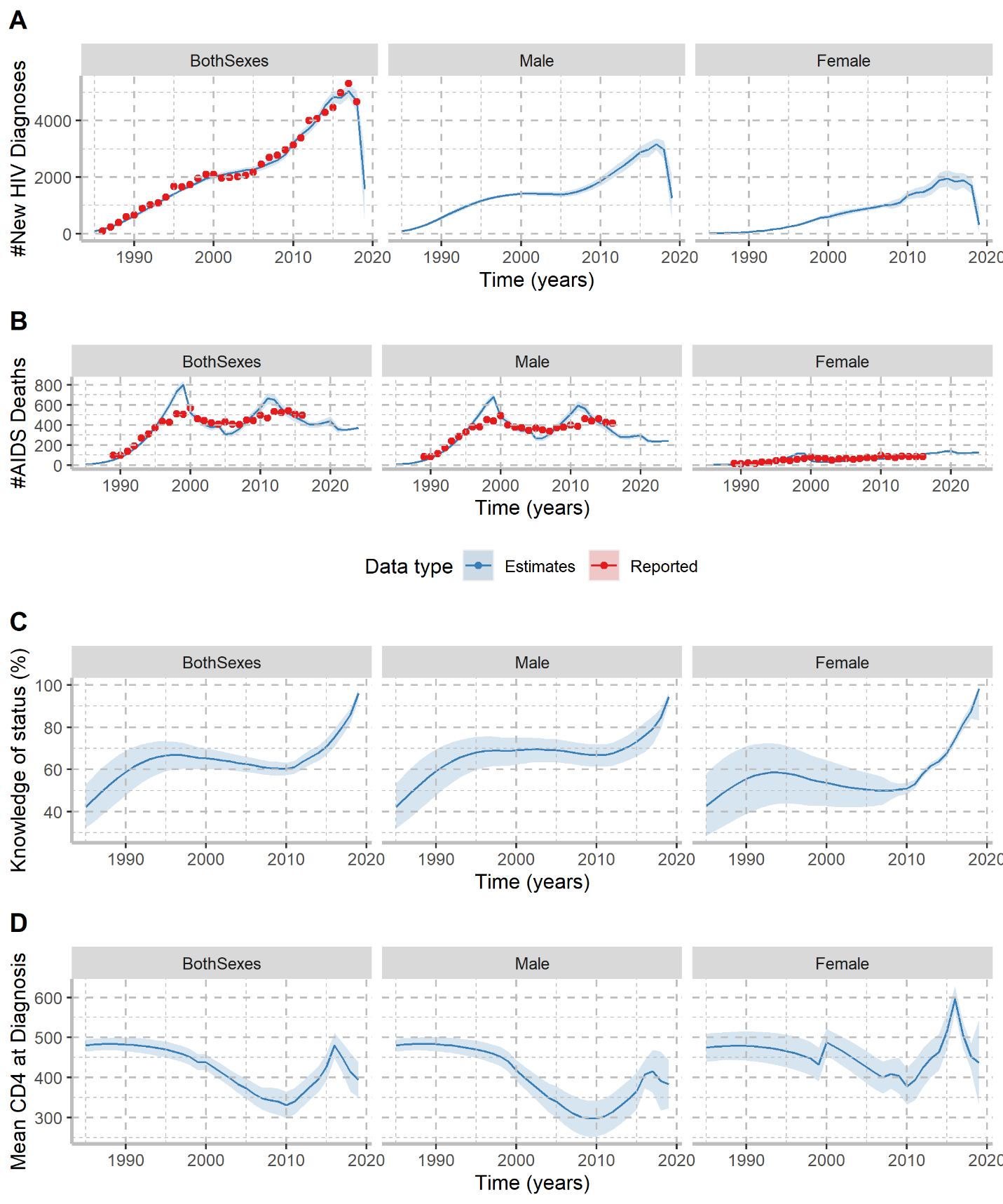
Figure S2:** Reported and estimated HIV indicators in Chile. A) Number of new HIV diagnoses, B) number of AIDS deaths, C) proportion of people living with HIV knowing their status and D) mean CD4 at diagnosis. The red dots represent numbers reported by the country, solid blue lines represent the posterior mode and shaded areas represent the 95%CI. Estimates were obtained using the model without key populations and incidence rate ratios were adjusted.

**Application 2: CSAVR with key populations in Bulgaria**

Bulgaria is one of the countries reporting the source of HIV infection to The European Surveillance System (TESSy) ^[^[^9-11^](#_ENREF_9)^]^. Published estimates of proportions of key populations and HIV prevalence among them were used to inform the priors ^[^[^12-16^](#_ENREF_12)^]^. All the four incidence curves were fitted in and AIC was used to choose the best fit. Figures S3 A and B show the estimated proportion of key populations and HIV of among those populations, respectively. The estimated proportions of MSM and MWID among men, were 0.94% (95%CI: 0.57%-1.55%), and 0.64% (95%CI: 0.47%-0.88%), respectively, and the estimation proportions of FSW and FWID among women were 0.52% (95%CI: 0.29-0.93%) and 0.12% (95%CI: 0.09%-0.17%), respectively. The HIV prevalence is relatively low, below 2% among MWID, FSW and FWID but increased from below 1% in 2010 to above 3% in 2019 among MSM. Figures S4 A, B, C and D show the reported and estimated numbers of new HIV diagnoses, AIDS deaths, and proportion of people living with HIV knowing their status, and mean CD4 at diagnosis and a function of sex and for key populations, respectively. The model seems to be adjusting to the data well and indicates that the overall knowledge of status has been increasing from about 50% in 2010 to about 75% in 2019. The mean CD4 at diagnosis appeared to be stable, around 400 since 2010 but was slightly higher among MSM at around 562 in 2019.

**
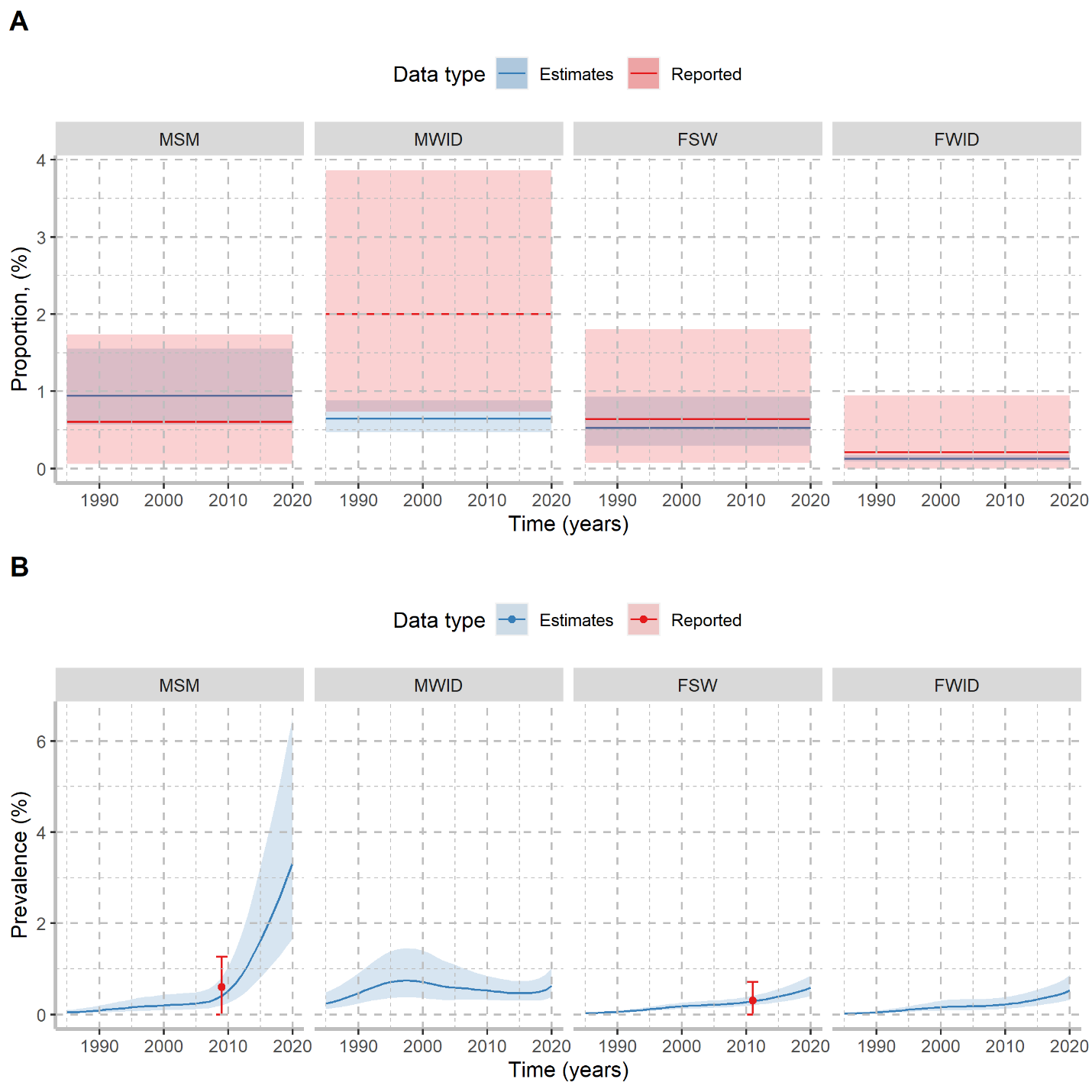
Figure S3:** Estimated proportion and HIV prevalence trends among key populations in Bulgaria.

**
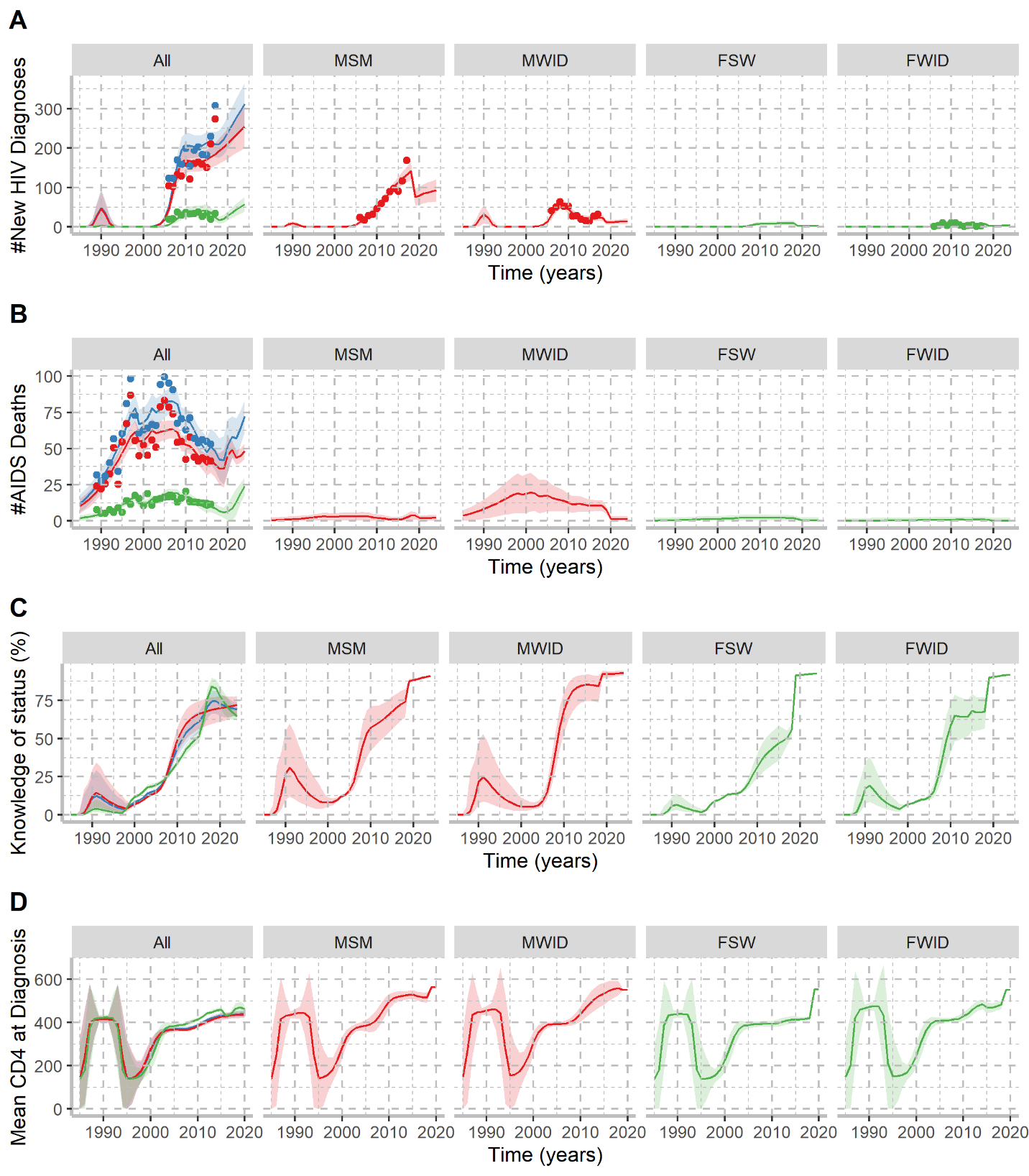
Figure S4:** Reported and estimated HIV indicators in Bulgaria. A) Number of new HIV diagnoses, B) number of AIDS deaths, C) proportion of people living with HIV knowing their status and D) mean CD4 at diagnosis. The dots represent numbers reported by the country; solid lines represent the posterior mode and shaded areas represent the 95%CI. Blue, red and green represent data for both sexes, men and women, respectively. Estimates were obtained using the model with key populations and incidence rate ratios were adjusted.

**Table S1 A: Prediction abilities of the model evaluated using the Continuous Ranked Probability Scores (CRPS, the lower the better) by region, excluding WCENA.**

| **UNAIDS Region** | **Incidence Model** | | | |
| --- | --- | --- | --- | --- |
|  | **Double Logistic** | **Logistic** | **Spline** | **r-Logistic** |
| ***AP*** | *1418.41* | *2580.03* | ***1129.65*** | *68742.38* |
| Australia | 207.96 | **154.74** | 172.60 | 1623.73 |
| Japan | **553.27** | 1627.24 | 553.30 | 8536.85 |
| New Zealand | **90.32** | 94.73 | 125.14 | **279.27** |
| Republic of Korea | 417.35 | 445.03 | **155.30** | 57025.59 |
| Singapore | 149.51 | **258.30** | **123.30** | 1276.94 |
| ***CAR*** | *769.25* | *1966.50* | ***652.31*** | *1011.70* |
| Bahamas | 99.07 | 478.84 | 98.77 | **89.13** |
| Barbados | **19.51** | 105.76 | 28.47 | 43.03 |
| Cuba | 242.12 | 949.19 | **126.11** | 546.74 |
| Trinidad and Tobago | 408.55 | 432.70 | 398.96 | **332.79** |
| ***EECA*** | *60673.74* | *86145.34* | *60660.17* | ***39991.38*** |
| Armenia | 205.14 | 687.92 | **132.38** | 697.05 |
| Belarus | 1464.77 | 2245.00 | **679.93** | 1158.60 |
| Georgia | **196.00** | 1276.18 | 251.94 | 494.60 |
| Kazakhstan | 1095.11 | 4365.49 | **927.80** | 4967.81 |
| Kyrgyzstan | 247.98 | 550.24 | **245.92** | 373.72 |
| Republic of Moldova | 261.22 | 557.83 | **213.27** | 538.42 |
| Russian Federation | **23.23** | 73.79 | 29.84 | 6712.03 |
| TFYR Macedonia | 56891.54 | 75640.52 | 57998.27 | **24562.35** |
| Uzbekistan | 288.73 | 748.38 | **180.81** | 486.81 |
| ***LA*** | *50614.57* | ***45160.34*** | *112263.31* | *93175.60* |
| Argentina | **1195.41** | 6574.79 | 3269.27 | 10047.73 |
| Brazil | 29339.53 | **18970.21** | 91432.84 | 64008.42 |
| Chile | **826.49** | 2618.44 | 2259.55 | 1418.08 |
| Colombia | 4948.69 | 5002.73 | 4853.63 | **4752.87** |
| Costa Rica | **179.25** | 355.51 | 234.36 | 246.27 |
| El Salvador | 548.53 | 996.38 | 532.11 | **496.09** |
| Mexico | 11796.99 | 8436.72 | **7613.29** | 9196.14 |
| Panama | 589.99 | 276.69 | **269.42** | 482.11 |
| Uruguay | 198.91 | 474.91 | 281.23 | **177.49** |
| Venezuela | **990.77** | 1453.95 | 1517.60 | 2350.40 |
| ***MENA*** | *4026.20* | *5381.47* | ***3423.79*** | *5917.07* |
| Algeria | 2071.30 | **1838.40** | 2546.62 | 2008.58 |
| Egypt | 1075.03 | 2074.70 | **177.51** | 2282.82 |
| Jordan | 28.73 | 27.33 | **13.85** | 13.23 |
| Kuwait | 40.28 | 45.03 | **38.07** | 300.00 |
| Lebanon | 242.77 | 122.90 | **77.78** | 250.20 |
| Oman | **94.37** | 96.58 | 111.04 | 119.23 |
| Qatar | 12.52 | **11.04** | 13.11 | 12.62 |
| Saudi Arabia | 382.32 | 850.42 | **371.31** | 395.58 |
| United Arab Emirates | 78.88 | 315.07 | **74.51** | 534.82 |

**Table S1 B: Prediction abilities of the model evaluated using the Continuous Ranked Probability Scores (CRPS, the lower the better) in WCENA**

| **UNAIDS Region** | **Incidence Model** | | | |
| --- | --- | --- | --- | --- |
|  | **Double Logistic** | **Logistic** | **Spline** | **r-Logistic** |
| ***WCENA*** | ***31070.82*** | *60861.97* | *51406.93* | *57487.43* |
| Austria | **78.70** | 182.75 | 108.28 | 483.71 |
| Belgium | 226.20 | 371.06 | **222.20** | 1939.47 |
| Bulgaria | 285.69 | 247.86 | **193.80** | 488.15 |
| Croatia | **34.56** | 117.96 | 92.85 | 47.49 |
| Cyprus | 41.81 | 57.57 | **28.65** | 143.44 |
| Czech Republic | 222.29 | 217.05 | 255.42 | **155.77** |
| Denmark | **59.83** | 78.03 | 85.14 | 72.73 |
| Estonia | 211.78 | 323.49 | **106.78** | 318.69 |
| Finland | **35.41** | 138.92 | 487.10 | 64.19 |
| France | 4790.30 | **4707.53** | 5851.38 | 6483.62 |
| Germany | **660.88** | 1081.48 | 3101.66 | 7647.53 |
| Greece | 396.50 | 475.61 | **221.86** | 1492.82 |
| Hungary | 388.09 | **103.63** | 414.25 | 224.88 |
| Iceland | 12.09 | **11.57** | 16.47 | 33.87 |
| Ireland | **69.45** | 106.41 | 85.17 | 685.55 |
| Israel | 258.47 | **245.10** | 294.41 | 161.18 |
| Italy | 3371.96 | 6223.54 | **2974.29** | 7676.58 |
| Latvia | **162.68** | 204.13 | 307.99 | 241.14 |
| Lithuania | 188.00 | 168.93 | 183.07 | **125.85** |
| Luxembourg | 14.35 | **12.56** | 18.16 | 21.93 |
| Malta | **22.30** | 62.93 | 23.61 | 173.67 |
| Netherlands | 633.90 | 1096.01 | **152.87** | 772.39 |
| Norway | **59.88** | 91.55 | 120.95 | 358.14 |
| Poland | 594.91 | **555.10** | 687.19 | 1728.49 |
| Portugal | 837.31 | 1386.99 | **665.57** | 1295.65 |
| Romania | 578.69 | 447.92 | 842.82 | **391.47** |
| Serbia | 83.71 | **74.54** | 113.57 | 144.12 |
| Slovakia | **46.40** | 136.57 | 100.06 | 1279.35 |
| Slovenia | 67.31 | 57.42 | **17.26** | 24.75 |
| Spain | **1972.48** | 10066.44 | 5808.73 | 7803.19 |
| Sweden | 68.39 | **54.49** | 100.30 | 1198.37 |
| Switzerland | **162.77** | 806.70 | 252.97 | 800.53 |
| Turkey | 2206.82 | 6159.53 | 2518.91 | **1385.18** |
| United Kingdom | **1437.55** | 4081.24 | 2491.69 | 2974.30 |
| United States of America | 10789.34 | 20709.34 | 22461.49 | **8649.22** |

**References**

1. Mahiane SG, Marsh K, Glaubius R, Eaton JW. **Estimating and projecting the number of new HIV diagnoses and incidence in Spectrum's case surveillance and vital registration tool**. *AIDS* 2019; 33 Suppl 3:S245-S253.

2. Mahiane SG, Marsh K, Grantham K, Crichlow S, Caceres K, Stover J. **Improvements in Spectrum's fit to program data tool**. *AIDS* 2017; 31 Suppl 1:S23-S30.

3. Bao L, Salomon JA, Brown T, Raftery AE, Hogan DR. **Modelling national HIV/AIDS epidemics: revised approach in the UNAIDS Estimation and Projection Package 2011**. *Sex Transm Infect* 2012; 88 Suppl 2:i3-10.

4. Li D-H, Fukushima M. **A modified BFGS method and its global convergence in nonconvex minimization**. *Journal of Computational and Applied Mathematics* 2001; 129(1-2):15-35.

5. Pham N. **Improved Nelder Mead’s simplex method and applications**; 2012.

6. Andrieu C, Thoms J. **A tutorial on adaptive MCMC**. *Statistics and computing* 2008; 18(4):343-373.

7. Akaike H. **A New Look at the Statistical Model Identification**. *IEEE Transactions on Automatic Control* 1974; (19):716-723.

8. Akaike H, Parzen E, Tanabe K, Kitagawa G. **Selected papers of Hirotugu Akaike***.* New York: Springer; 1998.

9. Prevention ECfD, Europe CWROf. **HIV/AIDS surveillance in Europe 2017–2016 data**. In: ECDC Stockholm; 2017.

10. Prevention ECfD, Europe CWROf. **HIV/AIDS surveillance in Europe 2018–2017 data**. *Copenhagen: WHO Regional Office for Europe* 2018.

11. Prevention ECfD, Europe CWROf. **HIV/AIDS surveillance in Europe 2019–2018 data**. In: ECDC Stockholm; 2019.

12. Degenhardt L, Peacock A, Colledge S, Leung J, Grebely J, Vickerman P, et al. **Global prevalence of injecting drug use and sociodemographic characteristics and prevalence of HIV, HBV, and HCV in people who inject drugs: a multistage systematic review**. *Lancet Glob Health* 2017; 5(12):e1192-e1207.

13. Marcus U, Hickson F, Weatherburn P, Schmidt AJ, Network E. **Estimating the size of the MSM populations for 38 European countries by calculating the survey-surveillance discrepancies (SSD) between self-reported new HIV diagnoses from the European MSM internet survey (EMIS) and surveillance-reported HIV diagnoses among MSM in 2009**. *BMC Public Health* 2013; 13:919.

14. Miller WM, Buckingham L, Sanchez-Dominguez MS, Morales-Miranda S, Paz-Bailey G. **Systematic review of HIV prevalence studies among key populations in Latin America and the Caribbean**. *Salud Publica Mex* 2013; 55 Suppl 1:S65-78.

15. Sabin K, Zhao J, Garcia Calleja JM, Sheng Y, Arias Garcia S, Reinisch A, et al. **Availability and Quality of Size Estimations of Female Sex Workers, Men Who Have Sex with Men, People Who Inject Drugs and Transgender Women in Low- and Middle-Income Countries**. *PLoS One* 2016; 11(5):e0155150.

16. Vandepitte J, Lyerla R, Dallabetta G, Crabbe F, Alary M, Buve A. **Estimates of the number of female sex workers in different regions of the world**. *Sex Transm Infect* 2006; 82 Suppl 3:iii18-25.
